# Flexible, portable and heatable non-woven fabric with directional moisture transport functions and ultra-fast evaporation†

**DOI:** 10.1039/d0ra03867a

**Published:** 2020-07-23

**Authors:** Jinhao Xu, Binjie Xin, Xuanxuan Du, Chun Wang, Zhuoming Chen, Yuansheng Zheng, Mengjuan Zhou

**Affiliations:** School of Textiles and Fashion Engineering, Shanghai University of Engineering Science Shanghai 201620 China xinbj@sues.edu.cn; College of Textiles, Donghua University Shanghai 201620 China; State Key Laboratory of Separation Membranes and Membrane Process, Tianjin Polytechnic University 300387 China

## Abstract

Compared with previous textiles possessing a hierarchical roughness structure for accelerating moisture evaporation, the use of Joule-heating to prepare heatable textiles is a more novel and useful way to achieve ultra-fast evaporation. Herein, we report an assembly strategy to create a functional non-woven (NW) fabric for directional moisture transportation and ultra-fast evaporation, ameliorating previous shortcomings. The resulting functional NW fabric reaches a sheet resistance of 1.116 Ω □^−1^, and the increased surface temperature (76.1 °C) induced by a low voltage (5 V) further results in an excellent ultra-fast evaporation rate (3.42 g h^−1^). Also, the moisture is transported to the outer surface of the designed fabric and spreads onto this surface. This desirable property can expand the contact area between sweat and the heatable fabric, further improving the evaporation efficiency, while maintaining the dry state of human skin. Generally, this functional textile with remarkable moisture management capabilities could be applied in winter outdoor sportswear to maintain human comfort.

## Abstract 2

Functional non-woven fabric with directional moisture transport and ultra-fast evaporation properties is demonstrated.

## Graphical abstract

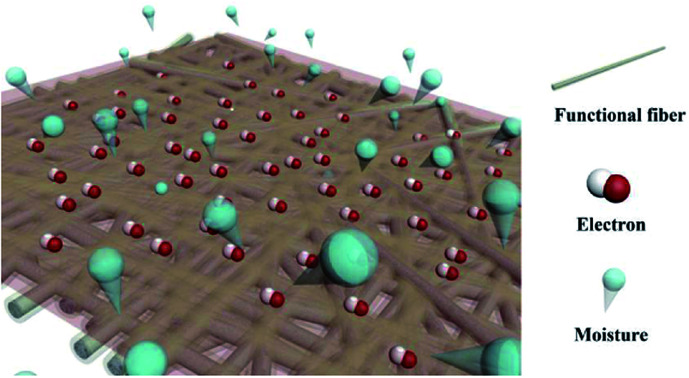

## 1. Introduction

The functions of directional moisture transportation and ultra-fast evaporation play a vital role in the comfort provided by clothing, thereby attracting increasing attention.^1–4^ Textiles with these functions can regulate microclimate comfort in particular environmental conditions and they are widely used in the fields of sportswear, overalls and uniforms.^5,6^ If sweat is impeded by apparel, and thereby adheres to the skin, it not only affects the thermophysiological functions, but also simultaneously causes dermatosis.^7,8^ Traditional textiles, such as cotton and nylon, absorb moisture through the wicking process and exhibit the same wetting behaviour on both sides of the fabric.^9^ This results in it being difficult to achieve desirable directional moisture transportation and ultra-fast evaporation. Therefore, it is necessary to develop new textiles with directional moisture transportation and ultra-fast evaporation at the same time.

Nowadays, to address these limitations, considerable approaches have been created to develop advanced textiles to provide superior moisture management. Miao *et al.*^10^ fabricated a trilayered fibrous membrane with a continuous, spontaneous, and directional water transport performance. Under the action of the wettability gradient, moisture was transported in the trilayered fibrous membranes and rapidly evaporated onto the surface with a hierarchical micro–nano scale roughness. Bing Dai *et al.*^11^ tailored a kind of Janus textile with conical micro-pores for wearing comfort. A simplified mathematical model was established in this work to depict the role of conical micro-pores in moisture management. As a result, it indicated that the conical micro-pores, the asymmetric hydrophilic pores from large to small (LTS) openings, can accelerate water transportation, while the evaporation rate was promoted by expanding the spread area of the water on the outer surface. Wang *et al.*^12^ reported a novel biomimetic micro–nano fibrous membrane based on Murray's law, which showed an antigravity directional water transport performance and quick drying times. In the resulting porous Murray membranes, a hierarchical structure consisting of multi-branching porous networks promoted the transportation of water from the inner layer to the outer layer, and provided a relatively large specific surface area to achieve ultra-fast evaporation rates. Although these studies exhibit excellent characteristics for sweat transportation and evaporation, they all accelerate moisture evaporation by roughening the surface, thereby increasing the surface area as much as possible. Compared with the aforementioned passive evaporation method, the heatable textile can achieve ultra-fast evaporation more aggressively. This positive sweat evaporation directly promotes a quick-drying performance and provides a novel path for maintaining wearing comfort.

The orientation motion of electrons generates Joule-heating,^13–16^ which is an effective method that is used to produce heatable textiles and has been widely used in daily apparels. For instance, Hsu *et al.*^17^ coated standard fabric with a conductive mixture slurry containing silver nanowires (AgNWs) and carbon nanotubes (CNTs). The resulting fabric can raise the temperature to 55 °C at a voltage of 1.5 V. Liu *et al.*^18^ deposited a thin silver film on the substrate with thermal evaporation for thermal management of the human body. Qiu *et al.*^19^ presented a composite heating fabric with a sandwich structure. A layer of carbon nanofibers embedded with various inorganic nanoparticles was sandwiched between two standard PET fabrics. The resulting fabric was suitable for using as a wearable heating textile in many fields. Nevertheless, few studies have used Joule-heating to accelerate moisture evaporation in textiles. Moreover, owing to the hydrophobic surface,^20–23^ moisture transport is restricted in most previous heatable textiles.

By comparing and analysing the previous studies, we propose that Joule-heating could realize the rapid evaporation of moisture on the surface of a textile, and hydrophilic surface treatment can further promote the evaporation and transportation of moisture. A non-woven (NW) fabric with functions such as directional moisture transport and ultra-fast evaporation has been developed using a four-step strategy. Initially, an ultra-thin silver (Ag) film composed of nanoparticles was deposited onto the surface of pristine NW as the essential heating elements. These heating elements can induce Joule heating, causing the moisture to evaporate rapidly in a positive manner. Additionally, the heating elements were covered with a hydrophilic coating, which is a mixture containing polyacrylonitrile (PAN) and silica nanoparticles (SiO_2_ NPs). To further enhance the hydrophilicity, the as-prepared NW fabrics were hydrolysed under weakly alkaline conditions. As a rough hydrophilic coating was formed on the surface, the inertness of NW to water was converted to an affinity for water, which significantly improved the wettability of the NW fabric to promote moisture transportation. Finally, to achieve directional moisture transportation, a polyvinylidene-fluoride (PVDF) coating was sprayed on one side of the hydrophilic NW to construct the wetting difference between the two sides. Consequently, the obtained functional NW had the capability of pulling the moisture out in a certain direction, followed by ultra-fast evaporation on the heated surface.

## 2. Experimental section

### 2.1 Materials

Polyethene (PE)/polyester (PET) composite NW (20 g m^−2^) fabric with an average fibre diameter of 16.8 μm was provided by Shanghai Fengge Non-woven Co., Ltd. A silver (Ag) target equipped in magnetron sputtering was purchased from Luoyang Lingshi New Material Technology Co., Ltd. Polyacrylonitrile (PAN, *M*_w_ = 90 000), flake sodium hydroxide (NaOH, 97%) and ethanol absolute (C_2_H_5_OH, 99.9%) were purchased from Aladdin Chemicals Co., China. Silica nanoparticles (SiO_2_ NP) with an average particle size of about 10–50 nm were purchased from Shandong Yousuo Chemical Technology Co., Ltd. PVDF (*M*_w_ = 400 000) was purchased from Arkema Co., France. Dimethylformamide (DMF, 99.5%) was of analytical grade, and was purchased from Sinopharm Chemical Reagent Co., Ltd China. The distilled water used in this work was obtained from a Heal-Force water purifying system. All of the analytical grade chemicals were used without further purification.

### 2.2 Preparation of functional non-woven fabric

The essential heatable element was prepared using a magnetron sputtering system (MSP-300C, Beijing Chuangshiweina Technology Co., Ltd). Before sputtering, the vacuum chamber was evacuated down to 9.5 × 10^−4^ Pa, and the distance between the target and substrate was fixed at 150 mm. The NW was fixed on the rotating platform at a speed of 10 rpm for uniform deposition. During the depositing process, high purity argon (≥99.5%) was introduced at a rate of 20 ml min^−1^, and the vacuum in the chamber was kept at 1.5 Pa. The Ag nanoparticles were deposited on both sides of the NW surface. Various sputtering powers and times were used to explore the optimal parameters. Detailed deposition parameters are shown in Table S1,† and the obtained samples were named NW-Ag.

A slurry for the coating was prepared by dissolving a specific concentration of SiO_2_ NP (3 wt%) and PAN (12 wt%) in DMF. A coating machine (XT-300SL, Shijiazhuang Xixiti Machinery Technology Co., Ltd) equipped with a Meyer rod (diameters: 400 mm) was employed. The Meyer rod was moved twice at different speeds throughout the coating process. When the first coating was applied, the movement speed was maintained at 10 mm s^−1^, so that the slurry was sufficiently dispersed on the surface of the fabric and then the movement speed was increased to 45 mm s^−1^ to promote uniform distribution of the slurry. The load mass was about 30 mg cm^−2^, and all coated samples were named CNW-Ag.

A certain amount of sodium hydroxide (10 g) was dissolved in a mixed solution (C_2_H_5_OH : H_2_O = 7 : 3 v/v, 100 ml). The CNW-Ag was dipped in a sodium hydroxide solution at 35 °C for 20 min to change it from moderately hydrophilic to super hydrophilic. After hydrolysing, the obtained samples were washed three times in distilled water to neutralize the pH value. Finally, the samples were dried at 50 °C in a vacuum and named HNW-Ag.

**Fig. 1:**
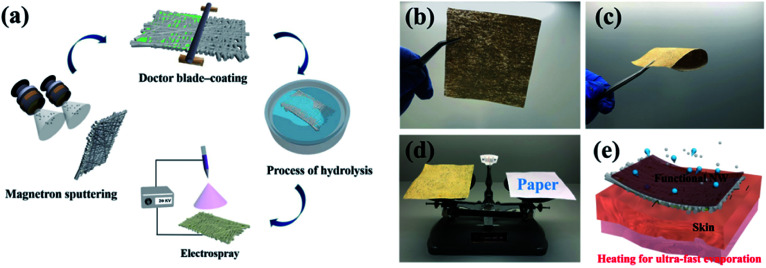
(a) A schematic illustration of the preparation of the fabricated NW fabric, (b)–(d) photographs of the fabricated NW fabric, the fabricated NW fabric under bending conditions, indicating the excellent flexibility, and the weight compared with paper, indicating the portability, and (e) a schematic illustration of the sweat output pathways of the human skin and rapid evaporation when covered with the fabricated NW fabric.

The PVDF was dissolved in DMF solvent followed by magnetic stirring for 8 h to prepare a PVDF solution (5 wt%). Afterwards, the as-prepared solution was electrosprayed onto one side of the HNW-Ag using an electrospinning device (RES-001 Rotary Dynamic). During the electrospraying process, the solution feeding rate was maintained at 0.003 mm s^−1^, a high direct current voltage of 20 kV was applied to the needle tip, and the distance between the needle tip and the collector was 15 cm. In the above mentioned mode, the spray times were set at 20, 60 and 120 min, respectively. All of the preparation details are shown in Fig. 1a.

### 2.3 Characterization

The surface morphology of the functional NW fabric was characterized using scanning electron microscopy (SEM, S4800 Hitachi Inc., Japan), and the elemental mapping was examined using energy-dispersive X-ray spectroscopy (EDS, 7593-H, Horiba Inc., Japan). The diameters of the fibres were calculated using image visualization software (Image J, NIH Image, Bethesda, MD, USA), and the thicknesses of each layer of the coating was calculated according to the change in the fibre diameter in the different processing stages.

Fourier transform infrared spectroscopy (FTIR, Perkin Elmer Inc., USA) and X-ray photoelectron spectroscopy (XPS, Thermo Scientific K-Alpha+) were employed to examine the chemical information of the NW, NW-Ag, CNW-Ag and HNW-Ag samples.

The sheet resistance values of the functional NW were measured using a four-point-probe resistance instrument (SZT-2C, Suzhou Tongchuang Electronics Co., Ltd). When the various mechanical deformations were exerted on the samples, the surface resistances were measured according to the AATCC test method 76-1995 with modifications. The copper sheets (50 × 10 mm) were fixed on both sides of the sample as the electrodes, and then the electrodes at both ends were connected with a multimeter. The resistances displayed by the multimeter (DT9801, Shanghai Yiji Electronic Technology Co., Ltd.) were recorded, and the samples were in a normal state before the mechanical deformations were applied. The resulting surface resistances are calculated using the following formula:^25^
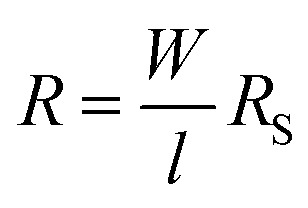
In which *W* represents the width between the two electrodes, *l* is the length of the sample between the two electrodes, and *R*_S_ is the resistance displayed by the multimeter. This was repeated at least three times for each sample. The water contact angles (WCAs) of the 5 μl droplets on the NW surface were measured using a contact angle tester (Beijing Jinshengxin Testing Instrument Ltd., China). During testing of the dripping diffusion, 200 μl deionized water was dropped onto one side of the NW fabric, and the diffusion process of water droplets on the surface was recorded using a digital camera.

The performance of Joule-heating was examined by coupling a DC power supply (RIGOL, DP832-A), a digital thermometer equipped with a K-type thermocouple (TES, TES-1310) and a thermal imager (FLIR). The DC power supply provided the voltage, the apparent changes in temperature were monitored by the thermocouple, and the thermal images can visibly exhibit the temperature distribution in the functional NW. When a DC power supply provided a voltage, the thermocouple monitored a significant change in the temperature, and the thermal image can visually display the distribution of the temperature in the functional NW. Furthermore, two copper sheets were employed to attach to each end of the samples as electrical contacts. The evaporation rates of water were tested based on GB/T21655.1-2008. A certain number of droplets (200 μl) were dropped onto the fabricated NW, and the weight was recorded every 2 min.^24^

The washing fastness of the conductivity and hydrophilicity was measured based on GB/T-3921-2008. Samples were washed at 40 °C with a 5 g L^−1^ detergent at a vibration frequency of 40 rpm using a constant temperature oscillator (BSD-TF270-370, Shanghai Boxun Industry Co., Ltd.). Each washing cycle was fixed within 30 min, and the samples were washed for 5, 10, 15 and 20 cycles, respectively.

The moisture permeability was tested based on GB/21655-2008, with modifications. The samples were fixed to the mouth of a transparent vessel containing the desiccant (calcium chloride, CaCl_2_), and the entire apparatus was placed in a stable environment (the temperature was 38 °C, and the relative humidity was 90%). The amount of water absorbed by the device was recorded over time. The performance of the air permeability was tested using an automatic permeability meter (YG461E-III, Ningbo Textile Instruments Ltd., China).

The mechanical performance of the functional NW was tested on a testing machine (YG006, Ningbo Textile Instruments Ltd., China) at a fixed stretching speed of 100 mm min^−1^. The dimensions of the samples were fixed parameters (20 × 150 mm), and the gauge of distance was set to 100 mm. The blending stiffness was measured using a FAST-2 bending tester, which was calculated using the following formula:BS = 9.81 × 10^−8^*WL*_B_^3^In which BS represents the blending stiffness, and *W* and *L*_B_ are the sample of the unit area quality (g m^−2^) and the blending length (mm), respectively.

## 3. Results and discussion

### 3.1 Design of flexible, portable, and heatable NW fabric for moisture management

Three criteria need be considered in the process of preparing functional NW fabrics: (i) the functional NW must be conductive in order to induce a Joule-heating effect at a specifically applied voltage; (ii) the functional NW is hydrophilic, which can accelerate evaporation by increasing the interface contact area through the spreading of water on the surface; and (iii) the surface energy difference needs to be constructed between the two sides of the functional NW. To satisfy the first criterion, the nano-metal particles are sputtered onto the pristine fibres, converting the insulating fibres into conductors. The second requirement is satisfied by applying a hydrophilic coating to form the desired hydrophilic polymeric matrix on the outermost layer. Finally, a polymer with a low surface energy is deposited on one side of the NW using single-side electrospray to construct the surface energy difference. Generally speaking, the functional NW fabric has the characteristics of flexibility, portability, and heat-generation (shown in Fig. 1b–d) owing to the distinct preparation process. In the designed fabric system, moisture can be smoothly transported from the inside to the outside and it then evaporates rapidly. Moreover, the performance of Joule-heating in the fabricated NW can not only promote moisture evaporation, but can also alleviate the final over-cooling of the post-exercise phase and keep the body warm after completing outdoor exercise at low temperatures (as shown in Fig. 1e).^9,23^

**Fig. 2:**
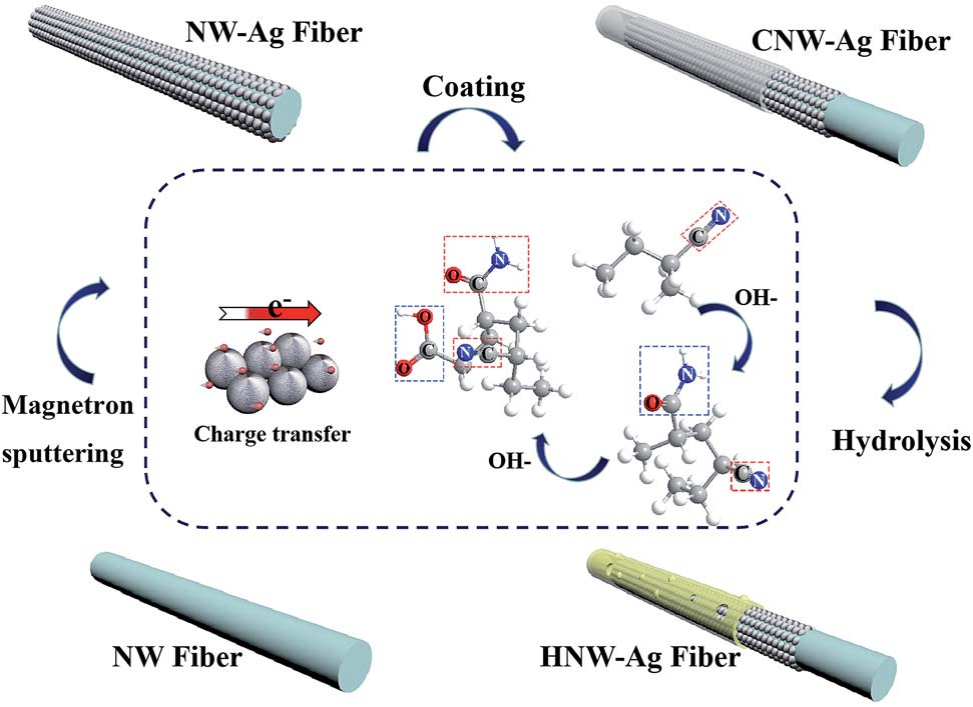
A schematic representation of the fabricated HNW-Ag.

Fig. 2 schematically exhibits the composition structure of the HNW-Ag. The pristine NW fibre acts as a mechanical support structure, an ultra-thin Ag film composed of nanoparticles is covered on the surface of the pristine NW fibre as a heating element, and a hydrolysed hydrophilic coating is located on the outermost surface to induce wetting behaviour.

**Fig. 3:**
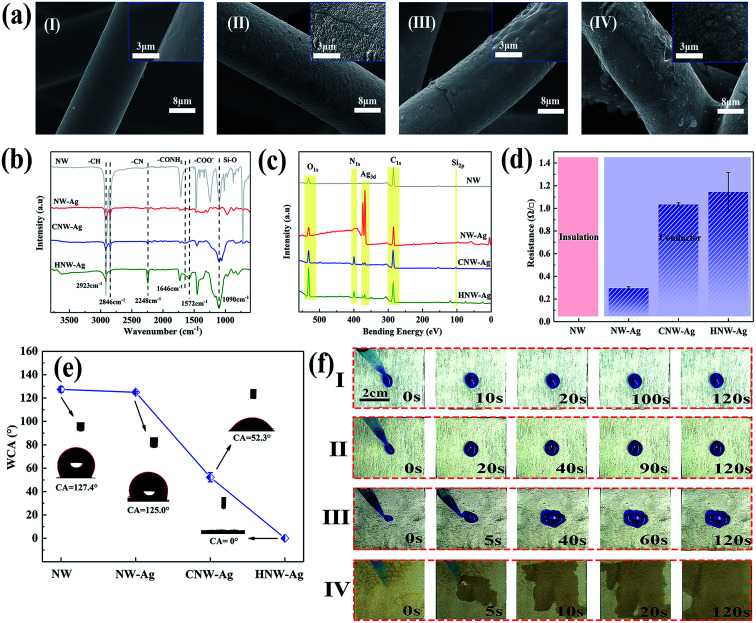
The behaviour of the fabricated HNW-Ag: (a) SEM images of pristine NW, NW-Ag, CNW-Ag and HNW-Ag samples, respectively, (b) and (c) FTIR and XPS spectra of the corresponding NW samples, (d) the sheet resistances of the corresponding NW samples, (e) the water contact angles and (f) the drip diffusion behaviours of the corresponding NW samples.

Fig. 3a depicts the surface morphologies of NW, NW-Ag, CNW-Ag and HNW-Ag, respectively, and the thickness of each layer of coating applied on the fibres is shown in Fig. S1 in the ESI.† The pristine NW is a smooth rod-like fibre structure with an average diameter of 16.5 μm. After magnetron sputtering, Ag nanoparticles with a particle size of approximately 200–300 nm are assembled onto the ultra-thin film with a thickness of 0.24 μm, which shows an uneven roughness. It was noted that the surface morphology of CNW-Ag changes from rough to flat owing to the dense coating on the surface. The morphology of the HNW-Ag fibre appears as both grooves and wrinkles again, and the thickness of the hydrophilic coating can be reduced from 1.25 to 0.97 μm, indicating that the coating is etched during alkali treatment. Fig. 3b illustrates the FTIR spectroscopy of each sample. Compared with the pristine NW fibre, the intensity of the absorption peaks in NW-Ag decrease owing to the Ag film coverage. During the conversion of CNW-Ag to HNW-Ag, the PAN coating undergoes a hydrolysis reaction in an alkaline environment, in which the –CN group is converted to –CONH_2_, then to a –COO^−^ group and NH_3_. Finally, the –COO^−^ groups in the macromolecule generate more hydrophilic –COOH groups in the water. In contrast with CNW-Ag, HNW-Ag shows a characteristic absorption peak for a carboxyl (–COO^−^) stretching band at 1572 cm^−1^, while the stretching vibration of the amide group (–CONH_2_) at 1646 cm^−1^ is weakened.^26,27^ In addition, the surface chemistry investigation was also analysed using XPS (shown in Fig. 3c). It was observed that significant asymmetrical peaks appear at 367.4 and 373.5 eV, respectively, indicating that the Ag film is successfully formed on the pristine fibre.^28^ Asymmetrical peaks intensities are decreased in CNW-Ag and HNW-Ag because the heating element is embedded in the hydrophilic coating. In addition, the surface chemistry evolution can also be obtained from the C 1s peaks (shown in Fig. S2†), which also confirms that PAN has been partially hydrolysed during the alkali treatment.

Fig. 3d demonstrates the sheet resistances of the corresponding NW fabrics.^30^ After sputtering, the NW fabric transforms from an insulator to a conductor, which reveals that the Ag film covering the surface of the fibre establishes an ideal conductive network.^29^ As shown in Fig. 3e, the WCAs of NW and NW-Ag can reach 127.4° and 125.0°, respectively, indicating that NW and NW-Ag have a strong hydrophobicity. Nevertheless, after the NW-Ag have been coated and the alkali treatment, the droplets can be wholly absorbed by HNW-Ag. The same result can also be obtained from the diffusion of the droplet on the surface of the corresponding NW fabrics (as shown in Fig. 3f). The HNW-Ag exhibits the desired affinity for water under the effects of the hydrophilic polymer matrix,^6^ confirming the successful conversion of the previous hydrophobicity to hydrophilicity.

### 3.2 Joule-heating in the NW fabric for ultra-fast evaporation performance

As the Ag film is embedded in HNW-Ag, it exhibits excellent conductivity, which can induce Joule-heating to promote ultra-fast evaporation. According to the basic kinetic theory,^31,32^ liquid evaporation can be represented as follows:
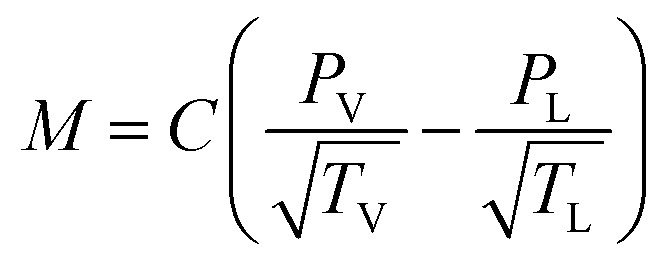
In which *M* represents the mass flux of interfacial evaporation, *P*_V_ and *T*_V_ are the pressure and temperature of the vapour on the liquid–air interface, respectively, and *P*_L_ and *T*_L_ are the pressure and temperature of the liquid on the liquid–air interface, respectively. *C* as a numerical value represents the corresponding liquid type. For ultra-fast evaporation, we hope to have a high *T*_L_ under stable ambient conditions. Therefore, it is designed that the *T*_L_ would be controlled by Joule-heating. According to the control variable experiments (see details in Fig. S3 and S4 and the “Supplementary discussion” section in the ESI†), the sputtering power and sputtering time of 60 W and 1000 s, respectively, are the most suitable parameters for the preparation of Ag films on the surface of fibres for inducing Joule-heating.

**Fig. 4:**
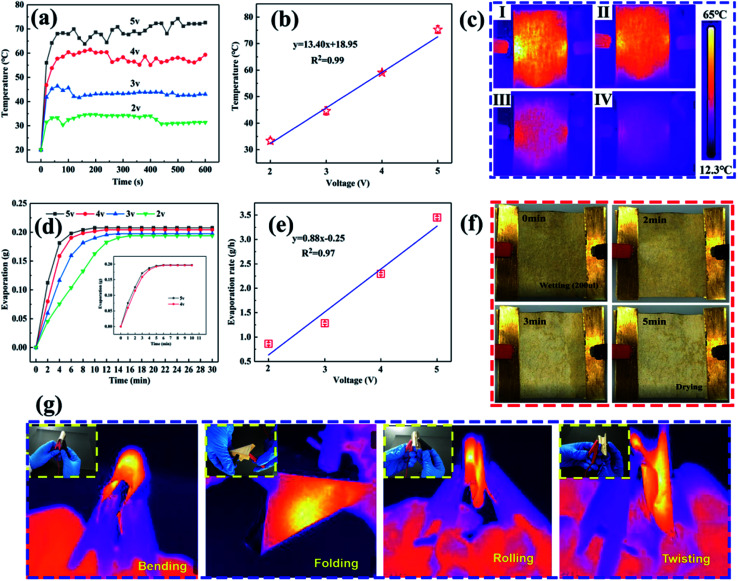
The behaviour of Joule-heating and ultra-fast evaporation in HNW-Ag: (a) time-dependent temperature performance during the Joule-heating of HNW-Ag (5 × 5 cm); (b) and (c) a summary of the average temperature and IR images at different voltages (2, 3, 4 and 5 V); (d) the water evaporation rates under different voltages; (e) and (f) a summary of the average water evaporation rates and physical pictures of water evaporation; and (g) IR images of HNW-Ag under bending, folding, rolling, and twisting conditions.

Fig. 4a exhibits the time-dependent temperature curves of the 5 × 5 cm samples at various voltages. During the experiment, the ambient temperature was maintained at 20 °C by the air conditioning system. It was noticed that once an additional voltage is applied, the surface temperatures of each sample rise rapidly and then remain within a relatively stable range. Owing to the increase of the Joule-heating power, the steady-state temperatures tend to be high with increasing additional voltages. This was calculated using the following equation: 
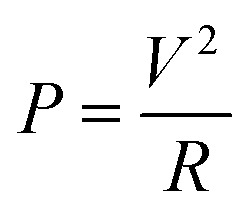
 in which *P* acts as the heating power, *V* is the applied voltage and *R* represents the total resistance of the sample.^13^ The same results can be visually observed in the infrared (IR) images (shown in Fig. 4c), in which the light yellow corresponds to the high-temperature zone, while the dark blue symbolizes the ambient temperature.

The average evaporation rates of HNW-Ag are 0.90, 1.35, 2.295 and 3.448 g h^−1^, respectively, at various voltages (2, 3, 4 and 5 V, as shown in Fig. 4d). It is apparent that the evaporation rates are continuously increasing, which proves that the Joule-heating can improve the mass flux of the interfacial evaporation, further realizing the top-speed drying of the NW fabric. The wet HNW-Ag can be completely dried within 5 min at an applied voltage of 5 V, which verifies the ultra-fast drying performance (as shown in Fig. 4f). As shown in Fig. 4b and e, the average temperatures and evaporation rates are summarized, and all the plots are fitted into lines. Interestingly, both surface temperatures and evaporation rates positively correlate with the applied voltages, and the fitted lines also depict the high *R*^2^ values, indicating a close correlation between the evaporation rate and applied voltage.

To investigate the flexibility of HNW-Ag after applying the voltage, various mechanical deformations, such as bending, folding, rolling, and twisting, which are analogous to human motions, were exerted on the samples. According to the resulting IR images, the samples depicted a stable heating performance, confirming the excellent flexibility and that they are suitable wearable fabrics. Fig. S5† exhibits surface-specific resistances of HNW-Ag with various mechanical deformations. Surface specific resistances of HNW-Ag under a normal state can be maintained at 1.02 Ω, after exerting various mechanical deformations on the samples, the resistances were found to be 1.08, 1.04, 1.03, and 1.03 Ω, which showed no visible change compared with the sample under the normal state. Therefore, the prepared fabric has a stable conductivity to support the generation of Joule-heating. Meanwhile, after washing for 600 min, the coating is still adhered to the surface of the fibres, and the measured sheet resistance and water contact angle remains at 1.19 Ω □^−1^ and 0° (as shown in Fig. S6 and S7†). These suggest the excellent adhesion durability of the heating elements and the hydrophilic coating.

### 3.3 Constructing a surface energy difference in NW fabrics for directional water transportation

**Fig. 5:**
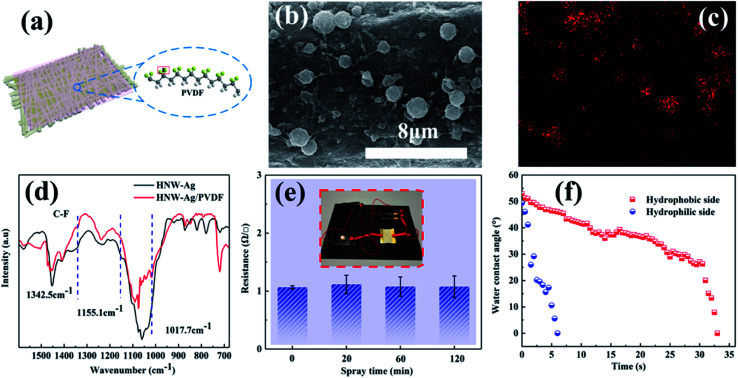
Asymmetric wetting behaviour of the functional NW fabric: (a) a schematic representation of the functional NW fabric; (b) SEM images of the functional NW fabric sprayed with PVDF; and (c) elemental mapping of F for the functional NW fabric. (d) FTIR spectrum of the functional NW fabric before and after spraying, (e) the sheet resistances of functional NW samples sprayed with PVDF for different times, and (f) changes in the WCA with time for water droplets on the functional NW fabric.

For construction of the surface energy difference in the fabricated NW fabrics to achieve directional moisture transport, one side of the functional NW was sprayed by PVDF (shown in Fig. 5a). Fig. 5b and S8† show the SEM images of the functional NW. It should be noted that a PVDF coating consisting of nano–microspheres is formed on one side of the functional NW. Furthermore, the loading of the electrosprayed side is adjusted by controlling the electrospraying time (0, 20, 60 and 120 min). Fig. 5c exhibits the element mapping of F on the electrosprayed side. It can be observed that the F element is randomly sprayed, and sufficiently covers the NW surface to form a thin layer. After the electrospraying, the stretching vibrations of C–F were observed at 1342.5, 1151.1 and 1017.1 cm^−1^ compared to HNW-Ag (shown in Fig. 5d),^12,33^ which confirms the formation of the PVDF layer on functional NW. As shown in Fig. 5e, the sheet resistances of the functional NW at different spraying times (20, 60, and 120 min) are 1.116, 1.078, and 1.076 Ω □^−1^, respectively. The deposited polymer does not increase the resistance of the functional NW compared to the pristine HNW-Ag, indicating that the capability to generate Joule-heating is still maintained in the functional NW.

Moreover, on the hydrophilic side, the WCAs reduce from 49.9° to 0° within 6 s, while on the opposite side, the WCAs reduce from 52.9° to 0° within 33 s (shown in Fig. 5f). Owing to the different surface energies on both sides of the functional NW, the droplet driving force *F* is generated as follows:^34,35^
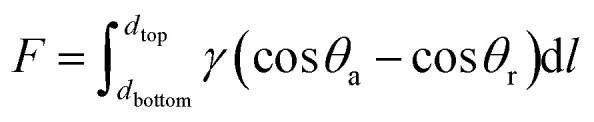
in which *θ*_a_ and *θ*_r_ are the advancing and receding contact angles of the droplet in the functional NW, respectively, and d*l* is the integral variable in the thickness direction from the region near the top (*d*_top_) to the region near the bottom (*d*_bottom_). Using the above mentioned formula, the driving force *F* promotes the transport of droplets from the low surface energy to a high surface energy, but impedes the transportation in the opposite direction. Therefore, the droplets penetrate the hydrophobic layer and are pulled toward the opposite side under the action of the driving force, but directly spread on the hydrophilic side.

**Fig. 6:**
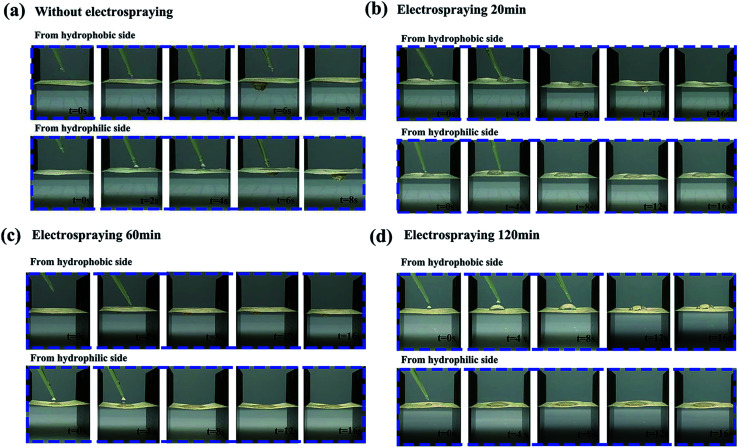
The behaviour of directional water transportation of the corresponding functional NW fabric sprayed with PVDF for: (a) 0; (b) 20; (c) 60; and (d) 120 min (water drops onto the upward hydrophobic side and hydrophilic side, respectively).

In order to investigate the effects of the PVDF loading on the directional water transport, the NW fabric with different PVDF loading values (shown in Table S3†) were prepared by controlling the electrospraying time, and the dynamic movements of the droplets (200 μl) were monitored by a set of digital photographs from a side view (shown in Fig. 6.). It is clear that the NW fabric without electrospraying shows a two-way water transport feature, meaning that the water can penetrate both sides of the NW under the conditions of good surface wettability. For the functional NW fabric with a PVDF loading of 1.6 g m^−2^ on one side, it behaves like a diode in a circuit, and the movements of the droplets in the functional NW are directly dependent on the side on which the droplets are dropped. When the PVDF coating is directed upward, droplets can be pumped through the sheet of functional NW. In contrast, when the functional NW is flipped, and the droplets are in contact with the other side, the droplets remain spherical and gradually spread horizontally rather than penetrate the fabric, successfully proving the performance of directional moisture transport in the functional NW. When the electrospraying time is prolonged to 120 min, and the weight loading of PVDF is 7.5 g m^−2^, it is worth noting that the NW fabric reveals a hydrophobic surface and the droplets remain hemispherical on the hydrophobic side without being transported to the opposite side. This behaviour can be ascribed to the strong hydrophobic force provided by the thick PVDF spray coating, which hinders the transportation of water in NW.^36,41^

**Fig. 7:**
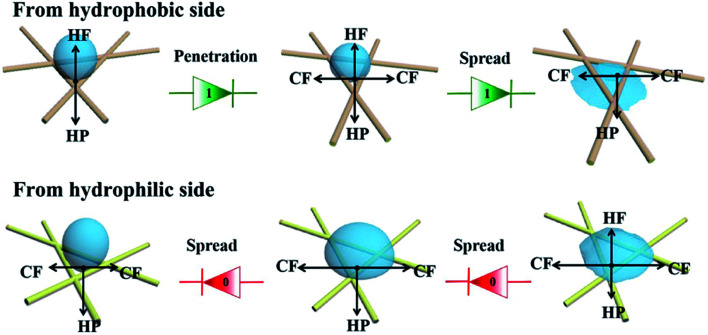
A schematic diagram illustrating the mechanism of directional water transport in the functional NW fabric.

Fig. 7 illustrates the mechanism of directional moisture transportation in the functional NW. Initially, we assumed that the droplets move in a positive direction from the PVDF coating side (hydrophobic side) to the HNW-Ag (hydrophilic side), while the opposite direction is negative. When the droplets are placed on the hydrophobic side, they are subjected to two opposite forces, hydrophobic force (HF) and hydrostatic pressure (HP), and retain a steady-state (Wenzel–Cassie state).^37,38^ Subsequently, this steady state is broken, and the droplets begin to move to the opposite side, owing to the action of the horizontal capillary force (CP) generated by HNW-Ag. Finally, the droplets further accelerate in the positive direction through the functional NW under the synergistic action of HP and CP.^39,40^ In contrast, when the droplets are dropped onto the upward hydrophilic side, they spread horizontally under the action of CP, rather than penetrate NW to the other side. Additionally, even though a small number of droplets penetrate the hydrophilic side and contact with the underlying PVDF coating, they can be prevented from making contact with the skin owing to the upward HF. In accordance with the discussion above, it is still impossible for droplets to be transported in a negative direction.

### 3.4 Comprehensive application analysis of the NW fabric

**Fig. 8:**
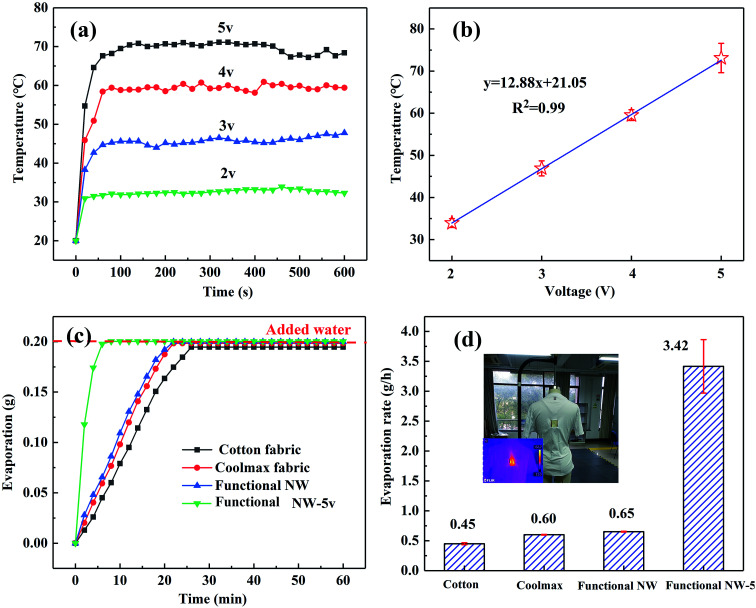
The Joule-heating and ultra-fast evaporation behaviour in the functional NW fabric: (a) time-dependent temperature performance during the Joule-heating of a functional NW sample (5 × 5 cm); (b) summary of the average temperatures at different voltages (2, 3, 4 and 5 V); (c) the time-dependent evaporation performance; and (d) the water evaporation rates of representative fabrics, and an image exhibiting a wearable functional NW sample.

Meanwhile, the Joule-heating and ultra-evaporation of functional NW after electrospraying were also characterized (shown in Fig. 8). The surface temperatures are 33.9 °C, 46.9 °C, 59.55 °C and 76.1 °C, respectively, confirming that the thin PVDF coating did not affect the Joule-heating performance of the obtained NW. Compared with traditional cotton fabrics and Coolmax fabrics, the functional NW fabric without an applied voltage has a better fast-drying property, owing to its rougher surface morphology and larger specific surface area.^13,36^ In particular, in the event of low voltages (5 V) being applied in functional NW fabrics, the average evaporation rate is sharply increased from 0.65 to 3.42 g h^−1^.

Interestingly, the functional NW possesses a decent mechanical property and excellent permeability, and remains in a soft state (shown in Fig. S9†). Therefore, these resulting performances indicate that the material as a functional element meets the requirements of a wearable textile. It is expected to be embedded in specific regions of sportswear, such as the trunk, back, abdomen and other areas in which sweat glands are densely distributed on the human body, to improve comfort.^41,42^

## 4. Conclusions

In summary, a flexible, portable and heatable functional NW fabric was successfully designed and fabricated to enhance the wearing comfort of sportswear. As Joule-heating is induced by surface metallization, the surface temperature of the functional NW is increased. Water can spread on the hydrophilic surface of the fabric, and the WCA can reach 0°, which can further accelerate the evaporation rate *via* increasing the interfacial contact area between the heated surface and the water. The evaporation rate is sharply promoted from 0.65 to 3.42 g h^−1^, which is superior to some commercial textiles. At the same time, a surface energy difference, leading to asymmetric wettability, is constructed in the obtained NW *via* spraying a PVDF coating on one side of the hydrophilic HNW-Ag. Directional water transportation occurs when the loading of the PVDF coating is 1.6 g m^−2^. Consequently, these promising results could provide a novel model to achieve excellent moisture management for winter outdoor sportswear.

## Conflicts of interest

The authors declare no competing financial interests.

## Supplementary Material

RA-010-D0RA03867A-s001

## References

[cit1] Cao M. Y., Xiao J. S., Yu C. M., Li K., Jiang L. (2015). Hydrophobic/Hydrophilic Cooperative Janus System for Enhancement of Fog Collection. Small.

[cit2] Zhao Y., Wang H., Zhou H. (2016). Directional Fluid Transport in Thin Porous Materials and its Functional Applications. Small.

[cit3] Wang Z., Zhao J., Bagal A., Dandley E. C., Oldham C. J., Fang T., Parsons G. N., Chang C. H. (2016). Wicking Enhancement in Three-Dimensional Hierarchical Nanostructures. Langmuir.

[cit4] Wang F., Annaheim S., Morrissey M., Rossi R. M. (2014). Evaporative Cooling Efficiency of One-Layer Tight-Fitting Sportswear in a Hot Environment. Scand. J. Med. Sci. Sports.

[cit5] Majumdar A., Mukhopadhyay S., Yadav R. (2010). Thermal Properties of Knitted Fabrics Made from Cotton and Regenerated Bamboo Cellulosic Fibres. Int. J. Therm. Sci..

[cit6] Rother M., Barmettler J., Reichmuth A., Araujo J. V., Rytka C., Glaied O., Pieles U., Bruns N. (2015). Self-Sealing and Puncture Resistant Breathable Membranes for Water-Evaporation Applications. Adv. Mater..

[cit7] Cheung S. S., Mclellan T. M., Tenaglia S. (2000). The thermophysiology of uncompensable heat stress: physiological manipulations and individual characteristics. Sports Med..

[cit8] Yetisen A. K., Manbachi H. Q. (2016). A Nanotechnology in Textiles. ACS Nano.

[cit9] Neves S. F., Campos J. B. L. M., Mayor T. S. (2017). Effects of clothing and fibres properties on the heat and mass transport, for different body heat/sweat releases. Appl. Therm. Eng..

[cit10] Miao D. Y., Zhan H., Wang X. F. (2018). Continuous, Spontaneous, and Directional Water Transport in the Trilayered Fibrous Membranes for Functional Moisture Wicking Textiles. Small.

[cit11] Dai B., Li K., Shi L. (2018). Bioinspired Janus Textile with Conical Micropores for Human Body Moisture and Thermal Management. Adv. Mater..

[cit12] Wang X. F., Huang Z., Miao D. Y., Zhao J., Yu J. Y., Ding B. (2018). Biomimetic Fibrous Murray Membranes with Ultrafast Water Transport and Evaporation for Smart Moisture-Wicking Fabrics. ACS Nano.

[cit13] Hazarika A., Deka B. K., Kim D. Y. (2018). Woven Kevlar® Fiber/Polydimethylsiloxane/Reduced Graphene Oxide Composite based Personal Thermal Management with Freestanding Cu-Ni Core-shell Nanowires. Nano Lett..

[cit14] Li X., Li Y., Guan T. T. (2018). Durable, Highly Electrically Conductive Cotton Fabrics with Healable Superamphiphobicity. ACS Appl. Mater. Interfaces.

[cit15] Allia P., Baricco M., Tiberto P. (1993). Joule-heating effects in the amorphous Fe_40_Ni_40_B_20_ alloy. Phys. Rev. B: Condens. Matter Mater. Phys..

[cit16] Zhang L., Baima M., Andrew T. L. (2017). Transforming Commercial Textiles and Threads into Sewable and Weavable Electric Heaters. ACS Appl. Mater. Interfaces.

[cit17] Hsu P. C., Liu X., Liu C. (2015). Personal Thermal Management by Metallic Nanowire-Coated Textile. Nano Lett..

[cit18] Liu Q., Huang J., Zhang J. (2018). Thermal, Waterproof, Breathable, and Antibacterial Cloth with a Nano-Porous Structure. ACS Appl. Mater. Interfaces.

[cit19] Qiu K. L., Elhassan A., Tian T. H., Yin X., Yu J. Y., Li Z. L., Ding B. (2020). Highly Flexible, Efficient, and Sandwich-Structured Infrared Radiation Heating
Fabric. ACS Appl. Mater. Interfaces.

[cit20] Choi B., Hong J. (2018). Highly Conductive Fiber with Waterproof and Self-Cleaning Properties for Textile Electronics. ACS Appl. Mater. Interfaces.

[cit21] He S., Chen Z. M., Xin B. J., Zhang F. L. (2019). Surface functionalization of Ag/polypyrrole-coated cotton fabric by *in situ* polymerization and magnetron sputtering. Text. Res. J..

[cit22] Gao L., Wang Y., Hu X. (2019). Cellular Carbon-Film-Based Flexible Sensor and Waterproof Supercapacitors. ACS Appl. Mater. Interfaces.

[cit23] Melikov A., Ivanova T., Stefanova G. (2004). Seat headrest-incorporated personalized ventilation: thermal comfort and inhaled air quality. Build. Environ..

[cit24] American Association of Textile Chemists and Colorists , Electrical resistivity of fabrics, AATCC Test Method 76-1995, 1996

[cit25] Micusk M. (2007). Conductive polymer-coated textiles-the role of fabric treatment by pyrrole-functionalized triethoxysilane. Synth. Met..

[cit26] Zhang G., Meng H., Ji S. (2009). Hydrolysis differences of polyacrylonitrile support membrane and its influences on polyacrylonitrile-based membrane performance. Desalination.

[cit27] Jin S. Y., Kim M. H., Jeong Y. G. (2017). Effect of alkaline hydrolysis on cyclization reaction of PAN nanofibers. Mater. Des..

[cit28] Bazant P., Kuritka I., Munster L. (2015). Microwave solvothermal decoration of the cellulose surface by nanostructured hybrid Ag/ZnO particles: a joint XPS, XRD and SEM study. Cellulose.

[cit29] He S., Xin B., Chen Z. (2018). Flexible and highly conductive Ag/G-coated cotton fabric based on graphene dipping and silver magnetron sputtering. Cellulose.

[cit30] Celle C., Céline M., Eléonore M. (2012). Highly flexible transparent film heaters based on random networks of silver nanowires. Nano Res..

[cit31] Luo Y. N., Ben W. (2019). Patterned Surfaces for Solar-Driven Interfacial Evaporation. ACS Appl. Mater. Interfaces.

[cit32] Carey V. P. , Liquid Vapor Phase Change Phenomena: An Introduction to the Thermophysics of Vaporization and Condensation Processes in Heat Transfer Equipment, Hemisphere Publishing, New York, 2nd edn, 2007

[cit33] Wang X., Wang Z. (2017). Tethering of hyperbranched polyols using PEI as a building block to synthesize antifouling PVDF membranes. Appl. Surf. Sci..

[cit34] Zheng Y., Bai H., Huang Z. (2010). Directional water collection on wetted spider silk. Nature.

[cit35] Ju J., Bai H., Zheng Y. (2012). A multi-structural and multi-functional integrated fog collection system in cactus. Nat. Commun..

[cit36] Wang H. J., Wang W. Y., Wang H. (2018). One-way Water Transport Fabrics based on Roughness Gradient Structure with No Low Surface Energy Substances. ACS Appl. Mater. Interfaces.

[cit37] Wu J., Wang N., Wang L. (2012). Unidirectional water-penetration composite fibrous film *via* electrospinning. Soft Matter.

[cit38] Xu J. H., Xin B. J., Chen Z. M. (2019). Preparation and characterization of multilayered superfine fibrous mat with the function of directional water transport. RSC Adv..

[cit39] Ahmed B. A., Mao D. Y., Mao A. (2018). Breathable and Colorful Cellulose Acetate-Based Nanofibrous Membranes for Directional Moisture Transport. ACS Appl. Mater. Interfaces.

[cit40] Wang H., Niu H., Zhou H. (2019). Multifunctional Directional Water Transport Fabrics with Moisture Sensing Capability. ACS Appl. Mater. Interfaces.

[cit41] Park S. J., Tamura T. (1992). Distribution of Evaporation Rate on Human Body Surface. Ann. Physiol. Anthropol..

[cit42] Brutin D., Starov V. (2018). Recent advances in droplet wetting and evaporation. Chem. Soc. Rev..

