# Supplementary material for: Flexible, portable and heatable non-woven fabric with directional moisture transport functions and ultra-fast evaporation
Source: RSC Adv. 2020 Jul 23;10(46):27512–22. doi: 10.1039/d0ra03867a (PMC9055594; doi:10.1039/d0ra03867a)
Supplement: RA-010-D0RA03867A-s001 [file RA-010-D0RA03867A-s001.pdf]

Electronic Supplementary Material (ESI) for RSC Advances.
This journal is © The Royal Society of Chemistry 2020

Supporting Information

Flexible, Portable and Heatable Non-woven Fabric with Functions of Directional Moisture Transport and Ultra-fast Evaporation

Jinhao Xu [1], Binjie Xin [1 *], Xuanxuan Du [1], Chun Wang [1 3], Zhuoming Chen [1], Yuansheng Zheng [1], Mengjuan Zhou [2]

1. School of Textile and Fashion Engineering, Shanghai University of Engineering Science, Shanghai 201620, China
2. Collage of Textile, Donghua University, Shanghai 201620, China
3. State Key Laboratory of Separation Membranes Membrane Process, Tianjin Polytechnic University 300387, China

* Correspondence to: Prof. Binjie Xin

School of Textile and Fashion Engineering, Shanghai University of Engineering Science, Shanghai 201620, China

Email addresses: xinbj@sues.edu.cn

Supplementary Tables

Table S1. The parameters in magnetron sputtering

Parameters	Range of variable			
Time	150s	300s	500s	1000s
	The sputtering power was fixed at 60w.			
Power	20w	40w	60w	100w
	The sputtering time was fixed at 1000 seconds.			

Table S2. The thickness of each layer coating applied on the fibers.

Sample	NW	NW-Ag (1000W, 60s)	CNW-Ag	HCNW-Ag
Average diameter(μm)	16.53±0.02	17.01±0.02	19.51±0.04	18.96±0.02
Layer thickness(μm)	-	0.24	1.25	0.97

Table S3. PVDF loading and water transport ability of NW fabric with different time durations of electrosprayinning

Electrospraying time (min)	PVDF loading (g/m^2)	Water transport ability
0	0	Two-way transport
20	1.6	One-way transport
60	4	Owe-way transport
120	7.5	None transport

Supplementary Figures

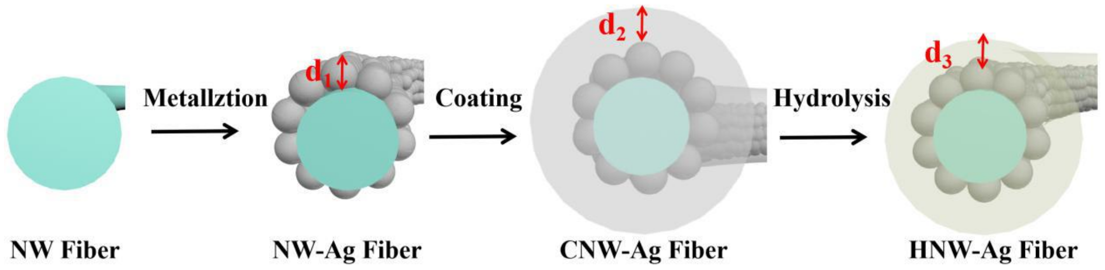

Figure S1.Schematic diagram of coating thickness variation.

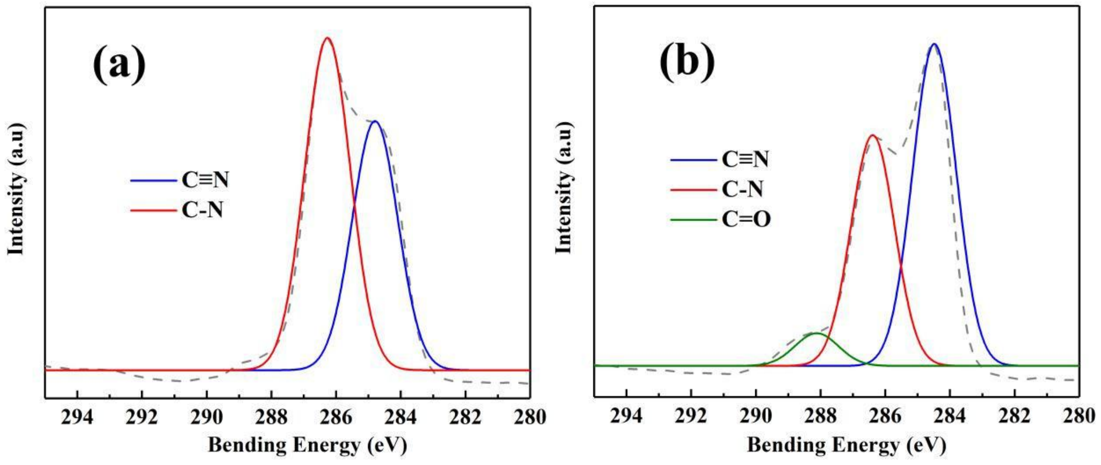

Figure S2. Angle resolved XPS spectra for C1s of (a) CNW-Ag and (b) HNW-Ag

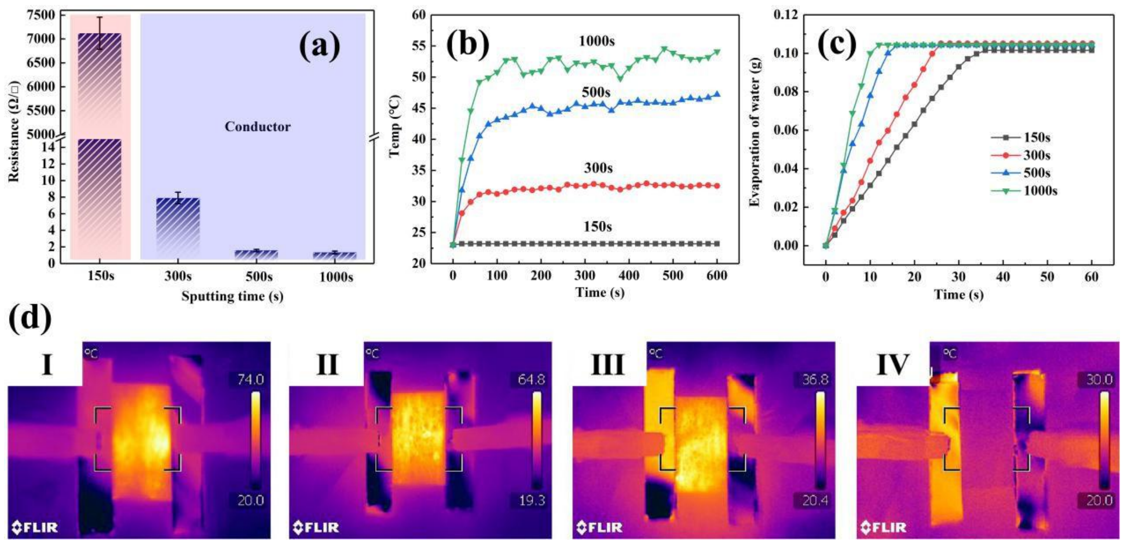

Figure S3. The behavior of Joule heating and ultra-fast evaporation with different magnetron sputtering time durations to the 2.5cm×2.5cm samples: (a) the sheet resistance, (b) time-dependent temperature performance, (c) water evaporation rate and (d) IR images of corresponding functional NW under the 3 voltages.

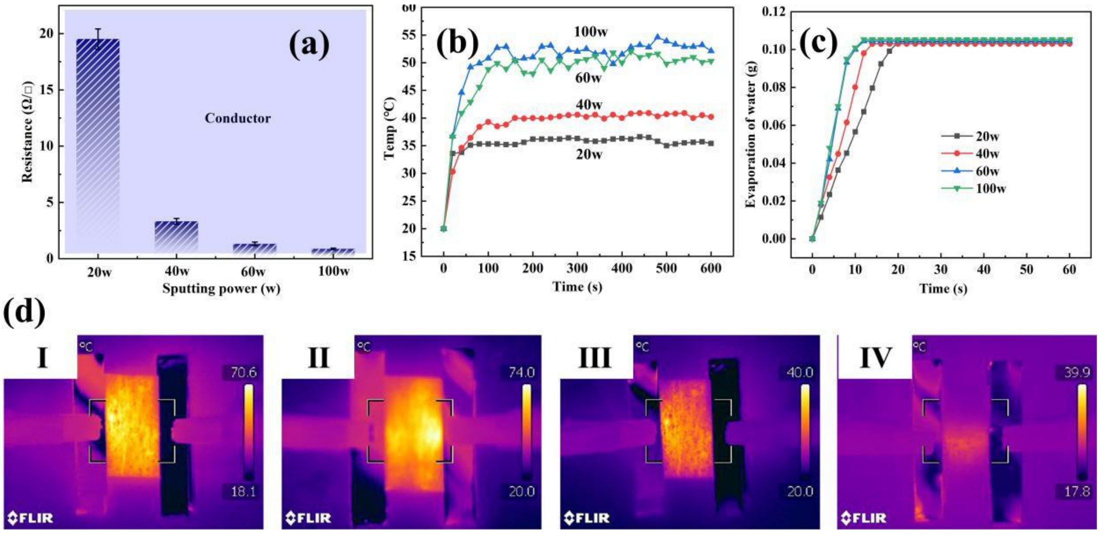

Figure S4.The behavior of Joule heating and ultra-fast evaporation with different magnetron sputtering powers to the 2.5cm×2.5cm samples: (a) the sheet resistance, (b) time-dependent temperature performance, (c) water evaporation rate and (d) IR images of corresponding functional NWs under the 3 voltages.

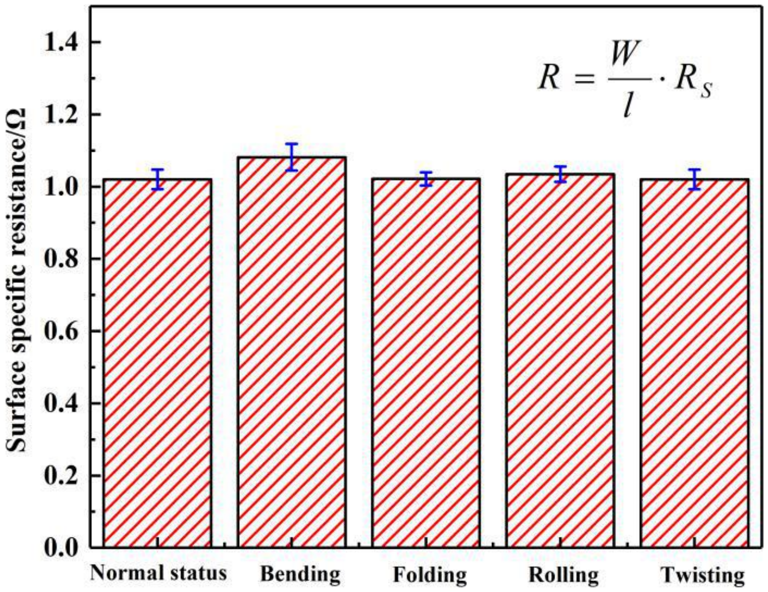

Figure S5. Surface specific resistances of HNW-Ag with various mechanical deformation.

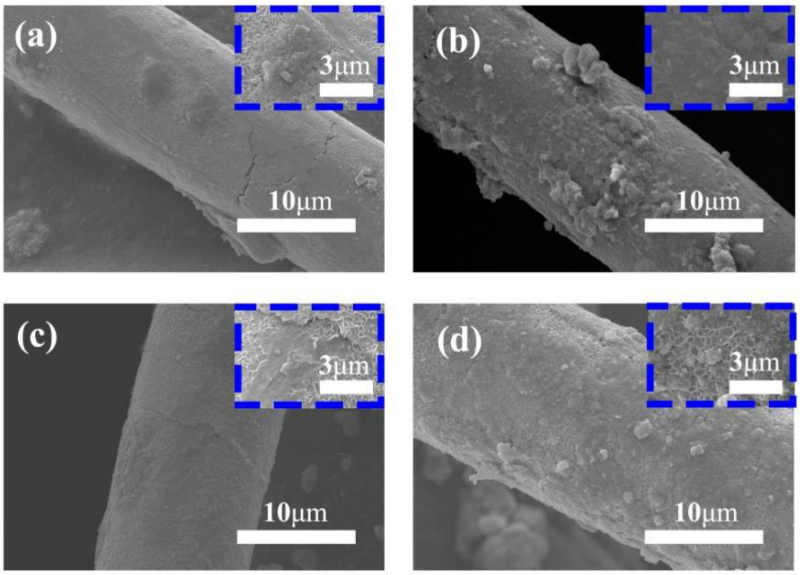

Figure S6. SEM images of functional NW after washing for (a) 150min, (b) 300min, (c) 450min and (d) 600min.

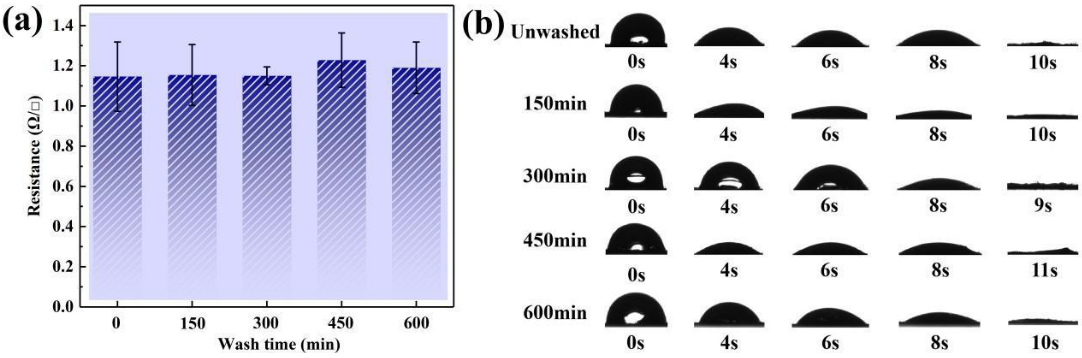

Figure S7. The sheet resistances and dynamic water contact angles of functional NW after washing for (a) 150min, (b) 300min, (c) 450min and (d) 600min.

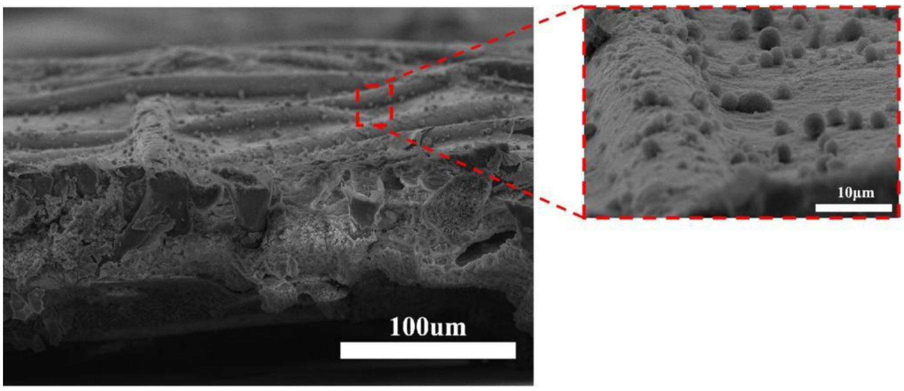

Figure S8. Cross-sectional SEM image of functional NW.

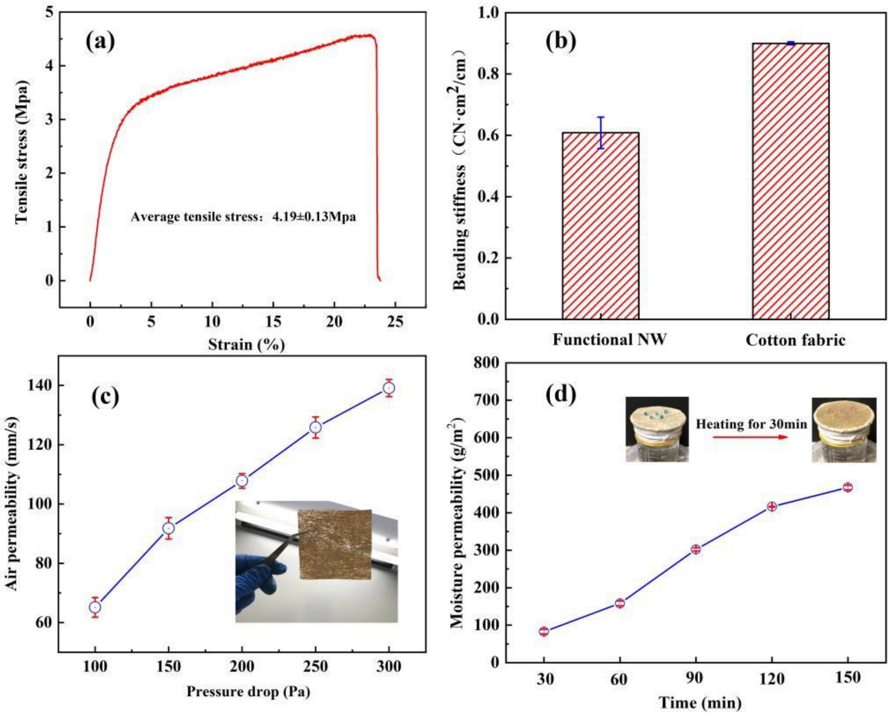

Figure S9. Wearable behaviors of functional NW: (a) tensile strength test, (b) bending stiffness of traditional cotton fabric and functional NW, and the performance of (c) air permeability test and (d) moisture permeability.

Supplementary Discussions

In order to determine the most suitable sputtering time and power in the process of magnetron sputtering, a series of HNW-Ag with different sputtering times and powers are fabricated, and the sheet resistance, Joule-heating performance and water evaporation rate are systematically characterized (shown in Figure S3 and Figure S4).

As shown in Figure S3a and Figure S4a, with the increase of sputtering time and power, the sheet resistances of HNW-Ag decrease obviously, and the resistance can reach the minimum (0.9Ω/□) when the sputtering power and sputtering time are 100w and 1000s, respectively. This is due to that a perfectly conductive network can be established after the Ag film are deposited on the NW fibers, and with the increase of sputtering power, the Ag particles are arranged more densely, which further increases the conductivity of the fabric

Figure S4b exhibits time-dependent temperature performance with different sputtering powers. When the sputtering power are 60W and 100W, respectively, both surface temperatures fluctuate around 55°C [1]. Since that arrangement of the distribution of Ag particles are already dense enough, the sheet resistances are very close when the sputtering power are 60W and 100W, respectively. Because the resistance values of the samples prepared at the two powers are close to each other, their Joule-heating performance intend to be same [2, 3]. The approximate surface temperatures make them consistent in their capability to evaporate moisture (shown in FS 4c). The same results can be visualized in FS 4d. However, in magnetron sputtering, low power can reduce the consumption of the target, thus the sputtering power and the sputtering time are 60w and 1000s, respectively is most suitable.

Reference:

1. S. He, B. Xin and Z. Chen, Flexible and highly conductive Ag/G-coated cotton fabric based on graphene dipping and silver magnetron sputtering, Cellulose, 2018, 25, 1530–1534.

2. Hazarika, B. K. Deka and D. Y. Kim, Woven Kevlar® Fiber/Polydimethylsiloxane/Reduced Graphene Oxide Composite based Personal Thermal Management with Freestanding Cu-Ni Core-shell Nanowires, Nano. Letters, 2018, 18, 6731-6739.

3. S. He, Z. M. Chen, B. J. Xin and F. L. Zhang, Surface functionalization of Ag/polypyrrole-coated cotton fabric by in situ polymerization and magnetron sputtering, Text. Res. J, 2019, 89, 884-4895.
